# Machine Learning Reveals Protein Signatures in CSF and Plasma Fluids of Clinical Value for ALS

**DOI:** 10.1038/s41598-018-34642-x

**Published:** 2018-11-05

**Authors:** Michael S. Bereman, Joshua Beri, Jeffrey R. Enders, Tara Nash

**Affiliations:** 1 Department of Biological Sciences, North Carolina State University, Raleigh, NC 27695 USA; 2 Department of Chemistry, North Carolina State University, Raleigh, NC 27695 USA; 3 Center for Human Health and the Environment, North Carolina State University, Raleigh, NC 27695 USA

## Abstract

We use shotgun proteomics to identify biomarkers of diagnostic and prognostic value in individuals diagnosed with amyotrophic lateral sclerosis. Matched cerebrospinal and plasma fluids were subjected to abundant protein depletion and analyzed by nano-flow liquid chromatography high resolution tandem mass spectrometry. Label free quantitation was used to identify differential proteins between individuals with ALS (n = 33) and healthy controls (n = 30) in both fluids. In CSF, 118 (p-value < 0.05) and 27 proteins (q-value < 0.05) were identified as significantly altered between ALS and controls. In plasma, 20 (p-value < 0.05) and 0 (q-value < 0.05) proteins were identified as significantly altered between ALS and controls. Proteins involved in complement activation, acute phase response and retinoid signaling pathways were significantly enriched in the CSF from ALS patients. Subsequently various machine learning methods were evaluated for disease classification using a repeated Monte Carlo cross-validation approach. A linear discriminant analysis model achieved a median area under the receiver operating characteristic curve of 0.94 with an interquartile range of 0.88–1.0. Three proteins composed a prognostic model (p = 5e-4) that explained 49% of the variation in the ALS-FRS scores. Finally we investigated the specificity of two promising proteins from our discovery data set, chitinase-3 like 1 protein and alpha-1-antichymotrypsin, using targeted proteomics in a separate set of CSF samples derived from individuals diagnosed with ALS (n = 11) and other neurological diseases (n = 15). These results demonstrate the potential of a panel of targeted proteins for objective measurements of clinical value in ALS.

## Introduction

Amyotrophic Lateral Sclerosis (ALS) is a progressive fatal disease with a median survival period of three years from symptom onset. It is the most frequent neurodegenerative disease of mid-life often striking individuals seemingly at random between 40 and 55 years of age^1,2^. There are no effective disease modifying therapeutics^3^ and the etiology of the disease remains largely outstanding with a small percentage of cases due to inherited gene mutations (5–10%)^4^. Moreover, no molecular biomarkers of diagnostic nor prognostic value exist. Diagnosis is often delayed 1–2 years from symptom onset while other confounding disorders are excluded and appropriate phenotypes (i.e. upper and lower motor neuron deterioration) present themselves. This qualitative disease assessment leads to misdiagnoses, prevents early intervention where future therapies are likely to be most effective, leads to both unnecessary expenditures (e.g., tests, surgeries) and patient anxiety. In addition, the ability to quantitatively assess disease progression and efficacy of therapeutic intervention in individuals diagnosed with ALS would be paramount in both the context of disease categorization and clinical trials.

Consequently the search for sensitive and specific markers in biological fluids is a highly active research area with enormous implications spanning ALS research, clinical care, and drug discovery. Studies have focused on small molecules, targeted proteins and modifications, oxidative stress markers, miRNAs, magnetic resonance imaging, and novel phenotypic markers^5–7^. Several studies have employed high-throughput proteomic approaches using mass spectrometry to discover novel markers. For the majority, these studies have been limited by small sample size^8^ and utilization of low peak capacity SELDI or MALDI-TOF techniques^9–12^. Ultimately these experimental designs limit the dynamic range of protein identifications, the accuracy of identifications, and or the precision of quantification. A recent study used liquid chromatography tandem mass spectrometry coupled with spectral counting based quantification was used to develop a classifier for separation of ALS and non ALS CSF samples with high sensitivity and specificity^13^.

Since CSF is proximal to site of injury, it is more likely to be enriched with biomarkers of ALS compared to plasma and is often the fluid of choice for ALS and other diseases of the central nervous system^13–18^. However, due to the ease of sampling and low probability of adverse effects, plasma remains an attractive yet challenging biological fluid for identification of ALS biomarkers. These challenges include the large dynamic range of plasma proteins^19^ and both intra- and inter-individual protein variability^20^. Despite these obstacles, it is critical to probe both fluids for diagnostic and prognostic markers using state of the art proteomic technologies. In addition the power of matched CSF and plasma samples could yield notable intra-individual protein comparisons which may lend insight into disease processes that are systemic or even more distal and support the hypothesis that ALS is a systems-wide disease that affects multiple organs^21–25^.

In this study nanoflow liquid chromatography coupled to high-resolution tandem mass spectrometry is used to investigate protein biomarkers in a set of matched plasma and CSF fluids derived from individuals diagnosed with ALS (n = 33) and healthy controls (n = 30). Intensity based relative quantification is used to identify differentially abundant proteins followed by evaluation of advanced machine learning algorithms to develop both diagnostic and prognostic models for use in ALS. Next we developed a targeted proteomic assay to investigate the specificity of two protein markers in a separate set of CSF samples from individuals with ALS and other neurological diseases. These data are then compared to targeted protein data from the healthy sample set. These results emphasize the power of a multi protein panel for clinical value in ALS.

## Materials and Methods

### Materials

Sodium deoxycholate (SDC) and urea were obtained from Sigma Aldrich (St. Louis, MO). Sequencing grade trypsin was from Promega (Madison, WI). Vivacon500® 30 kDa molecular weight cut off (MWCO) spin filters were purchased from ThermoFisher Scientific (Waltham, MA). Pierce top 12 abundant protein depletion spin columns were from ThermoFisher (#85164). HPLC grade acetonitrile, methanol, and water were from Burdick & Jackson (Muskegon, MI). Pico-frit columns were purchased from New Objective (Woburn, MA), and reversed phase ReproSil-Pur 120 C-18-AQ 3 µm particles were purchased from Dr. Maisch (Ammerbuch-Entringen, Germany). High purity nitrogen gas was purchased from Machine & Welding Supply Co (Raleigh, NC).

### Methods

#### Sample Preparation

De-identified plasma and CSF samples were obtained from the Northeastern Amyotrophic Lateral Sclerosis Consortium (NEALS) sample repository. Samples were prepared/analyzed following the agreement between the Bereman Laboratory and NEALS which focused on identification of protein biomarkers in plasma and CSF.

For plasma fluid, the manufacturer’s protocol for protein depletion was followed. However for CSF fluid, the amount of depletion material used was explored. Pooled CSF fluid was subjected to protein depletion with the following volumes of manufacturer depletion material: 45, 65, 85, and 105 µL in quadruplet. The number of protein identifications, efficiency of protein depletion, reproducibility, and cost were used to choose the optimal volume of material (85 µL). After depletion, protein concentration was quantified using a commercial BCA assay. All samples were normalized to the lowest total protein concentration by dilution with buffer. Samples were digested using established laboratory procedures and a modified filter aided sample preparation method. Trypsin was added at a 1:50 enzyme/protein ratio and digestion proceeded for 4 hours at 37 °C with agitation.

To control for variability and minimize any bias in the measurements, CSF and plasma samples were prepared using a randomized block design. All CSF or plasma samples were depleted of abundant proteins on the same day. Samples were then allocated to one of three cycles for sample digestion in efforts to minimize the variability in age, sex, and disease status across each group. CSF and plasma samples were prepared separately and each cycle contained approximately 20 samples. Within each cycle, samples were assigned a random number in order to blind the scientist to disease status throughout the entire sample preparation and database search.

#### LC MS/MS

Nanoflow LC MS/MS was performed using a 120-minute gradient ramp from 100% A (98/2 H2O/ACN 0.1% formic acid) to 40% B (100% ACN 0.1% formic acid). A 5 min wash (80% B) was followed by a 10 min column equilibration (100% A). Peptides were loaded directly on column at a flow rate of 400 nl/min. Peptides were separated at a flow rate of 300 nl/min using a 30 cm self-packed column. Data were collected using a top 12 data-dependent acquisition method on a quadrupole orbitrap (QE-Plus, Breman Germany). A resolving power @ m/z 200 of 70,000 and 17,500 were used for MS1 and MS2 scans, respectively. Automatic gain control was 1e6 and 1e5 for MS1 and MS2 scans respectively. Dynamic exclusion was set to 20 seconds to avoid repeated interrogation of abundant species and peptide match was set to ‘preferred’. A quality control bovine serum albumin digest was run every fifth injection to ensure proper LC-MS/MS reproducibility^26^. QC data were uploaded to Panorama using Panorama AutoQC^27^, and data showed retention time and full width at half-maximum median CV of 0.9% and 14.6%, respectively throughout the experiment. Parteo analysis of the cumulative sum control chart^28^ showed 2 and 1 outliers in peptide retention time and full width at half-maximum, respectively. The plasma data set yielded a median CV 1.6%, and 18.4% in retention time and full width at half-maximum, respectively. Parteo analysis identified 2 and 0 outliers in peptide retention time, full width at half-maximum, respectively. Careful examination of these outlier runs yielded no clear indicator of special cause variation in either dataset. All quantitative proteomics data used in these analyses are available in Supplemental File 1. All raw data collected for this study have been uploaded to the Chorus LC-MS/MS repository, project #1439.

#### Database Search

Database searches were first conducted using Proteome Discoverer 1.4 and the Sequest hyper-threaded algorithm. Protein data were searched against the SwissProt human proteome database (88,421 sequences, 2014). Cysteine carbamidomethylation was searched as a static peptide modification, and methionine oxidation was searched as a dynamic peptide modification. This search was used as an initial screen of sample quality to ensure a reproducible number of proteins were identified across all samples. One CSF sample was then removed from further analysis due to extremely low number of IDs. Label free quantitation was then performed in MaxQuant^29^ using the fast LFQ algorithm and the LFQ minimum ratio count set to 1^30^. Modifications for the MaxQuant search were the same as described *vide supra* with the addition of N-terminal protein acetylation. All other search parameters were left as default.

#### Targeted Protein Method Development & Analysis

Peptide surrogates for the two targeted proteins of interest were determined using an empirical refinement approach^31^. Peptides were initially filtered based on uniqueness, abundance, and chromatographic performance. We then investigated 24 hour peptide stability in the autosampler, optimized digestion times, an evaluated the necessity for protein depletion. All measurements were performed in triplicate. Two peptides were chosen for each protein and stable isotope labeled peptides (^13^C_6_^15^N_2_ lysine or ^13^C_6_^15^N_4_ arginine) were synthesized (New England Peptide, Gardner MA). A separate set of CSF samples from individuals diagnosed with ALS (n = 11) and disease controls (n = 15) were acquired from NEALS biorepository. A set of 12 healthy csf samples were randomly selected from the cohort used for the discovery investigations in addition to 3 new healthy samples and prepared alongside the two new sample sets. Sample preparation proceeded in a similar fashion as described *vide supra*. A mixture of SIL peptides were spiked just prior to digestion. A notable difference was protein depletion was not performed. Samples were analyzed using parallel reaction monitoring on a Q-Exactive HF and data analysis were performed in Skyline. A permutation test (n = 10,000) was performed to assess the difference in the median normalized peptide response amongst groups. A permutation test was used due to the significant deviation of the distribution of normalized peptide response from normality. The Skyline data has been uploaded to Panorama www.tinyurl.com/ALSMarker.

#### Univariate Statistics

CSF and plasma protein data were then separately imported into Perseus^32^. Protein abundances were log_2_ transformed. Proteins with more than 25% missing values in both groups (ALS and healthy) were removed from further analysis. Remaining missing values were then imputed with a random number selected from a normal distribution with a width of 0.3 and shifted 1.8 standard deviation units down from the mean abundance of each sample. The difference in the mean protein abundance between ALS and healthy samples was evaluated using a two tailed t-test. Multiple hypothesis testing correction was performed in Perseus using a permutation (n = 250) based FDR method.

To evaluate intra-individual protein correlation in plasma and CSF, two tests were performed. The intersection of all proteins that were identified in both CSF and plasma that met the describe filters was obtained. First simple linear regression was performed and the probability that the slope of the regression line was significantly greater than 0 was determined. To gain further confidence of significance, the Pearson correlation coefficient of each protein abundance between CSF and plasma in ALS versus healthy was calculated. A distribution of correlation coefficients was created between each permutation of the plasma (n = 10000) and the original CSF protein abundance. The p-value for significance was calculated by determining the number of values in the permutation distribution that were greater than the absolute value of the original Pearson correlation coefficient. This number was divided by the number of permutations (n = 10,000) and multiplied by 2 (two-tailed).

#### Multivariate Statistics

Data were imported into RStudio (v1.0.143). Several packages were used for multivariate analysis of the data including Applied Predictive modeling, e1071, carett, pROC, plyr and several other embedded packages. For classification, an unsupervised feature selection approach was utilized to choose 5 proteins. The protein list was first filtered by significance (q < 0.05) and then by correlation. If two proteins were correlated (Pearson coefficient > 0.6) then the protein with the lowest average correlation among all other proteins was retained. Finally the remaining top five most significant proteins were chosen for classification. Four different commonly used machine learning algorithms were evaluated including linear discriminant analysis, random forests, support vector machines, and generalized linear models. Data were first centered and scaled. Then five-fold repeated (n = 50) cross-validation was performed on the data using each method. For the GLM model, the logit link function was utilized. To ensure optimal performance both the cost parameter (2^−2^, 2^−1^, 2^0^…2^12^) and the number of randomly selected predictors at each split (m_try_ = 2:5) for the svm and random forest classifier were tuned, respectively. The sigma parameter for the radial basis function used in the svm was held constant and its predicted value^33^ (σ = 0.0333). Performance was evaluated using the accuracy, sensitivity, specificity, area under receiver operating characteristic curve (AUC) and Cohen’s Kappa statistic^34^. Since each model was trained and tested on identical subsets of data, a paired t-test was used to statistically assess model performance on the resampled data sets^35^.

Multiple linear regression was used to develop a model of prognostic value. The ALS functional rating score was used as a metric to gauge disease progression. Feature selection was similar as described vide supra with one change. After removing proteins that correlated (Pearson coefficient > 0.6), best subset selection was performed in which every combination of 1 to 7 (out of 16) protein variables were selected for the model. Models were evaluated by the adjusted r-squared value, *Mallows* C_p_^36^, and the *Bayesian information criterion*^37^. The fit of the final model was assessed by qualitative evaluation of the residuals versus fitted plot, density and QQ plots of the residuals.

For the plasma classifier, a supervised feature selection technique called recursive feature elimination^38^ was utilized to identify which proteins to include in the model. Three models were evaluated including linear and nonlinear support vector machines and random forests. The protein list was first filtered by correlation. If two proteins were correlated (Pearson coefficient > 0.6) then the protein with the lowest average correlation among all other proteins was retained. Data were then centered and scaled. Feature selection coincided with the model building process such as to encompass the variability of feature selection in the final results. Using a repeated (n = 5) 10 fold cross-validation approach, the model was first constructed using all 122 proteins with 90% of the data. Protein features were then ranked based on performance and subset sizes ranging from 1:25, 30, 40, 50, 60, 70, 80, 90, and 100 proteins were evaluated on the hold out sample set (10%). This inner-loop was repeated 9 additional times and then the whole process was repeated 5 times. The average area under the receiver operating characteristic curve, calculated from the resampled data sets (n = 50), was plotted as a function subset size for each model type to identify the optimal size. A paired t-test was used to statistically assess each optimized model’s performance on the resampled data sets^35^.

#### Pathway Analysis

Proteins found to be significant in the cerebrospinal fluid (p < 0.05) were submitted to Ingenuity Pathway Analysis. We followed specific guidelines for using the hypergeometric test to evaluate pathway enrichment including the submission of an empirical background protein database^39^. The database was created by using all of the proteins that were detected in at least two samples. P-value and Z-score were used to evaluate pathway enrichment. Protein interaction networks were created using the stringAPP application within Cytoscape^40^.

## Results and Discussion

**Figure 1:**
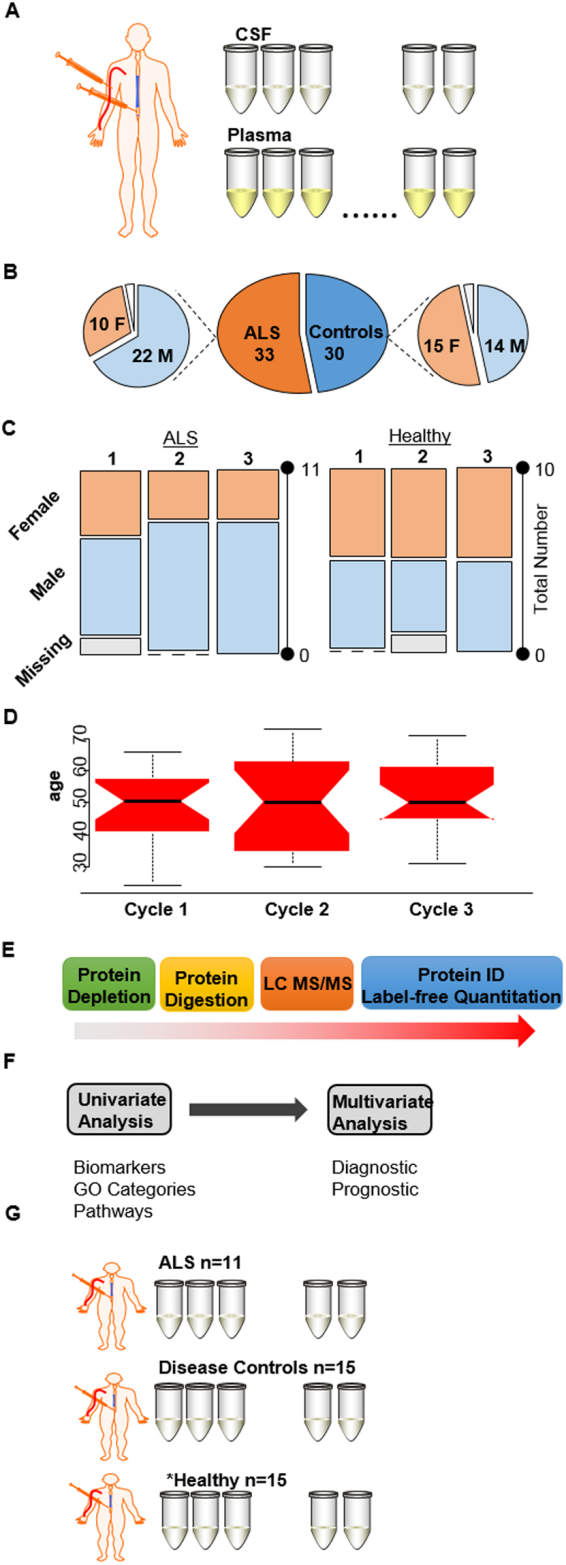
An overview of the sample set and experimental design. (**A**) Matched plasma and CSF samples derived from individuals diagnosed with ALS and healthy controls were obtained from the NEALS biorepository. (**B**) Pie charts of the number of males and females in the ALS and healthy sample set. (**C**) Mosaic plots describing the characteristics of the three cycles used for sample preparation. (**D**) Box plots of the age distribution in each cycle. (**E**) Samples were depleted of abundant proteins, digested using standard laboratory procedures, and analyzed by LC-MS/MS followed by protein identification and label free quantitation. (**F**) A combination of univariate and multivariate techniques were used to identify biomarkers, investigate perturbed pathways, and develop diagnostic and prognostic models. (**G**) Set of samples used for targeted proteomic experiments. *While the ALS and disease controls were unique to the targeted experiment, the majority of the heathy samples were the same in both experiments. Figure was partially created using images purchased in the PPT Drawing Toolkits-BIOLOGY Bundle from Motifolio, Inc.

Figure 1 describes the sample cohort and the overall workflow of the study. Matched de-identified plasma and CSF samples derived from healthy and individuals diagnosed with ALS were obtained from the Northeastern Amyotrophic Lateral Sclerosis (NEALS) Consortium biorepository. We received samples from 33 patients with ALS of which 66% were males. Our control set consisted of 30 individuals that were considered healthy (Fig. 1B). Samples were divided into 3 cycles for sample preparation and LC MS/MS analysis as shown in Fig. 1C. Sex, age, and disease status were blocked to minimize measurement bias. Each cycle consisted of 21 samples: 11 ALS and 10 controls. Based on the 95% confidence interval, the difference in the median age amongst the 3 cycles was insignificant (Fig. 1D).

Samples were subjected to abundant protein depletion and then digested using standard laboratory procedures. Peptide mixtures were analyzed by LC-MS/MS and label free quantitation was performed using the LFQ intensity calculated from MaxQuant. A combination of univariate and multivariate statistics were then used to identify biomarkers, gain insight into the perturbed molecular pathways and develop both diagnostic and prognostic models.

### Univariate Analysis

**Figure 2:**
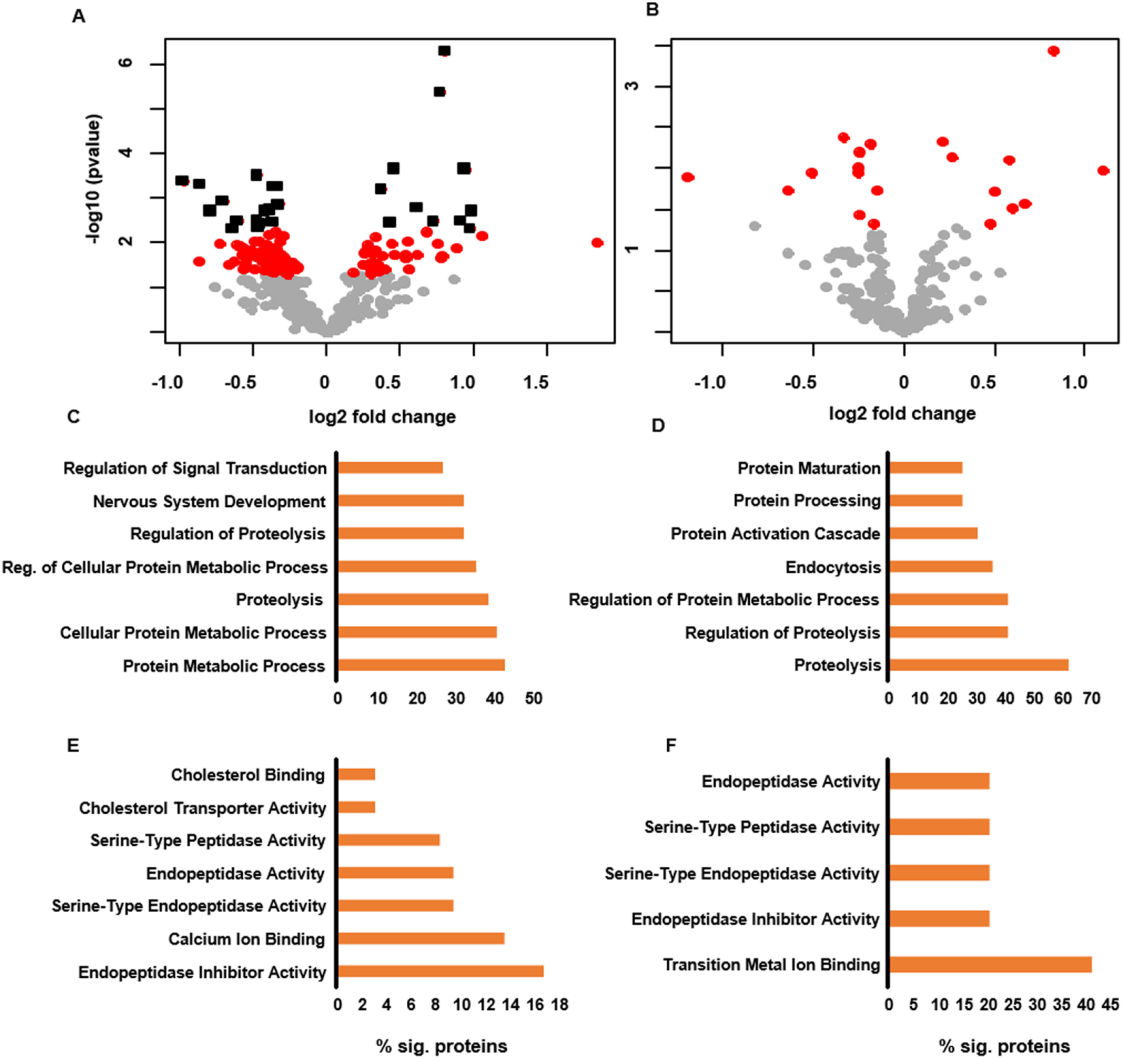
Volcano plots of the −log_10_ (p-value) versus the log_2_ fold change of proteins in ALS versus control for (**A**) CSF and (**B**) plasma fluids. Points colored gray, red, black indicate proteins with a p-value > 0.05, p-value < 0.05, and q-value < 0.05, respectively. GO analysis of biological processes and molecular function in CSF (**C** and **E**) and plasma fluids (**D** and **F**).

Figure 2A and B display volcano plots of proteins quantified in CSF and plasma between ALS and healthy controls respectively. A larger number of significant proteins (p < 0.05) were identified in the CSF compared to the plasma samples (118 vs. 20). The small number of significant proteins found in the plasma samples is most likely due to its distal nature, large dynamic range of plasma proteins^19^ and both intra- and inter-individual protein variability^20^. After adjustment for multiple hypothesis testing (q < 0.05), 27 and 0 proteins were identified as differentially abundant between healthy and ALS in CSF and plasma, respectively. A complete list of proteins quantified in CSF and plasma can be found in Supplemental File 1. Table 1 lists the top 10 most significant proteins (based on p-value) identified in CSF and plasma. Using the Ingenuity Knowledge Base, several of the most significant proteins in CSF and plasma have been previously associated with one or more neurodegenerative diseases including ALS. Gene ontology analysis revealed proteolysis as a conserved molecular function amongst the significant proteins quantified which corresponded to protein metabolism as a common biological process in both fluids. These findings could support a more systemic role for these processes in ALS.

**Table 1:** A list of the top 10 most significant proteins found in **A** CSF and **B** plasma fluids.

gene name	protein name	p-value	q-value	fold change	neurodegeneration
**A**					
CHI3L1	Chitinase-3-like protein 1	5.0E-07	0.000	0.80	ALS,H,A
SERPINA3	Alpha-1-antichymotrypsin	4.0E-06	0.002	0.77	A,P,FTD,ALS,H
APOD	Apolipoprotein D	2.1E-04	0.011	0.45	A,H,P
CHI3L2	Chitinase-3-like protein 2	2.2E-04	0.008	0.93	A
APP	Amyloid beta A4 protein	3.0E-04	0.010	−0.48	A,P,FTD,ALS
FAT2	Protocadherin Fat 2	4.1E-04	0.013	−0.98	n/a
CBLN1	Cerebellin-1	4.8E-04	0.014	−0.87	X
CPE	Carboxypeptidase E	5.3E-04	0.014	−0.36	X
CNTN2	Contactin-2	5.3E-04	0.012	−0.34	X
CFB	Complement factor B	6.2E-04	0.014	0.37	A
**B**					
PRG4	Proteoglycan 4	3.7E-04	0.068	0.83	n/a
SERPINA6	Corticosteroid-binding globulin	4.3E-03	0.410	−0.33	n/a
CFH	Complement factor H	4.8E-03	0.311	0.21	A
SERPINC1	Antithrombin-III	5.2E-03	0.254	−0.18	A
CP	Ceruloplasmin	6.4E-03	0.254	−0.24	A,H,W
APOB	Apolipoprotein B-100	7.4E-03	0.246	0.27	VD
HBA1	Hemoglobin subunit alpha	8.0E-03	0.231	0.58	n/a
GSN	Gelsolin	9.9E-03	0.254	−0.25	ALS
IGHV3-23	Ig heavy chain V-III region TIL	1.1E-02	0.244	1.10	n/a
PEPD	Xaa-Pro dipeptidase	1.1E-02	0.232	−0.51	n/a

X = General motor difficulties P = Parkinson’s disease H = Huntington’s disease A = Alzheimer’s disease VD = Vascular Dementia n/a = Not associated with neurodegenerative disease in the Ingenuity Knowledge Base.

**Figure 3:**
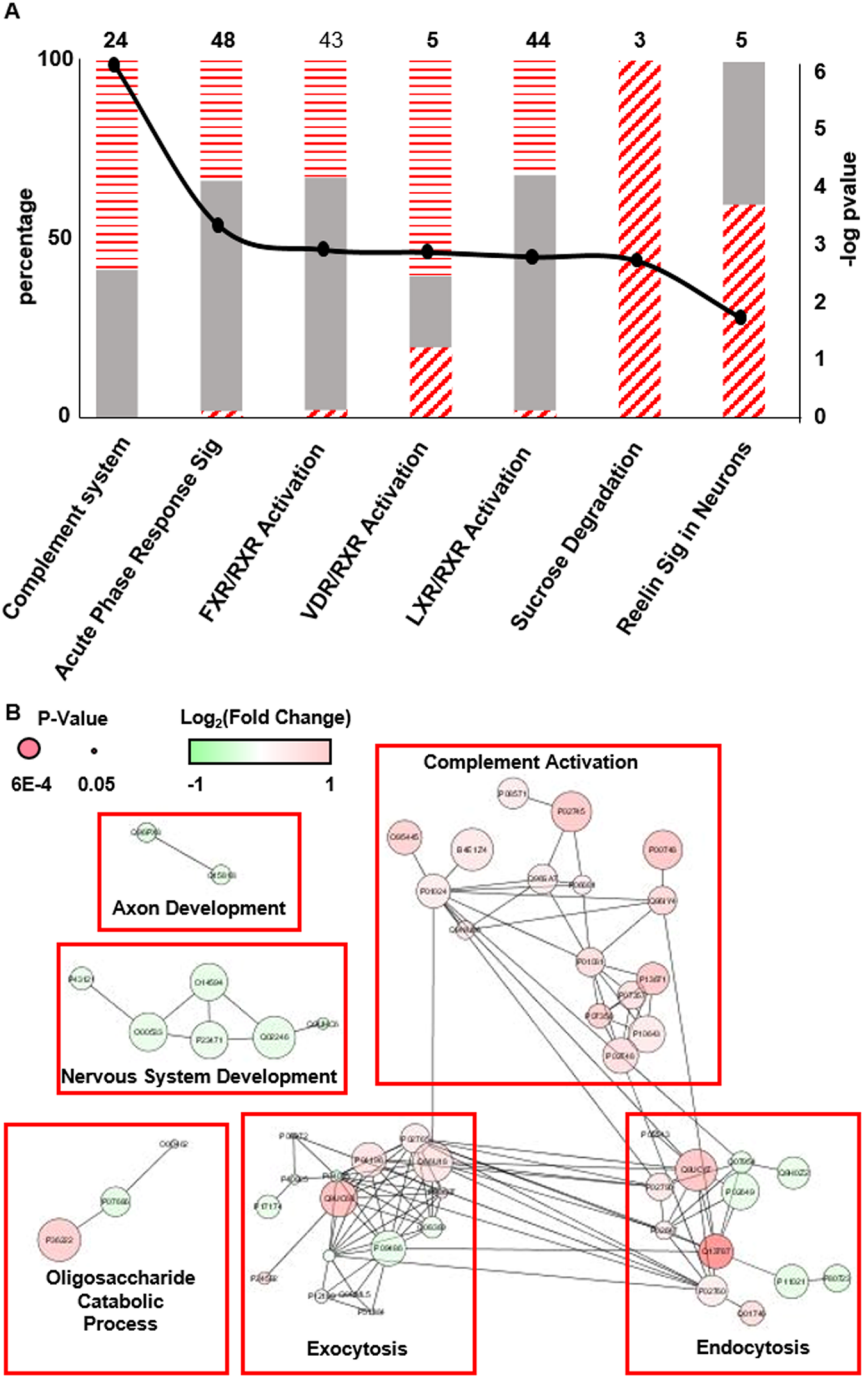
(**A**) A stacked bar chart of significantly enriched pathways derived from the differential proteins in the CSF data. Gray solid bars represent proteins that were not detected as differentially abundant. Horizontal and diagonal dashed bars represent proteins that are up- and down regulated, respectively. Left axis is the percentage of proteins detected in that pathway (top number) as unchanged or different. Right axis displays the significance of the enrichment. (**B**) Interaction network analysis of differentially abundant proteins. The size of the circle is proportional to the significance (i.e., p-value) while the shade is indicative of the fold change. Clusters of proteins were isolated and subjected to GO analysis to determine biological function.

Figure 3A shows the significantly (p ≤ 0.05) enriched pathways from the differentially abundant proteins in CSF. Also displayed are the percentage of significant proteins in each pathway that were found to be up- or down-regulated. Several pathways were found to be significant which have previously been shown to be perturbed in ALS patients. These include activation of the complement system, a component of innate immunity, which has been shown to be activated both in cerebrospinal fluid, spinal cord, and motor cortex of ALS patients^41–43^ as well as rodent models of ALS^44^. Other pathways notably affected were several pertaining to the retinoid X receptor, RXR, including FXR/RXR, VDR/RXR, and LXR/RXR activation pathways. Retinoid signaling pathways, which are critical for neural development and neural regeneration^45^ and have been shown to promote neural regeneration in adult rodents^46^, have previously been observed to be disrupted in ALS^47^.

Figure 3B shows visualization of known protein interactions. Protein data were analyzed within Cytoscape^40^ version 3.6.0 using the stringAPP plugin for protein interaction network analysis. Groups of interacting proteins were isolated and individually subjected to gene ontology analysis using DAVID 6.8^48^ to determine biological function. Groups deemed significant (Benjamini-corrected p-value ≤ 0.05) were assigned to the biological function with the lowest p-value. Multiple protein group functions were identified as significant which have previously been demonstrated to be perturbed within ALS patients, including activation of the complement system, nervous system and axon development, and bulk transport functions (i.e., exocytosis and endocytosis). In particular, exocytotic and endocytotic functions play a role which are fundamental to many processes known to be disrupted in ALS development, including neurotransmitter release and membrane signal regulation^49^ as well as ejection of misfolded protein from the cell. Interestingly, it has been found that misfolded mutant SOD1 can cause misfolding of wild-type SOD1^50^, and that this conversion may be propagated between cells through transmission of mutant SOD1 from cell to cell through an exocytotic process^51^.

While mass transfer of small molecules and to a lesser extent peptides and proteins can occur across the blood cerebral spinal fluid barrier^52^, the two fluids are believed to be independently regulated^53^. It was hypothesized that protein abundances that were correlated between CSF and plasma fluids but not healthy controls may indicate a role for these proteins outside the central nervous system. We took the intersection of the significant proteins found in CSF data with the proteins identified in plasma regardless of statistical significance. Complement component 7 (p = 0.03) and retinol binding protein 4 (p = 0.003) displayed a statistical positive correlation in plasma and CSF in ALS patients yet an insignificant negative correlation (p > 0.3) in fluids from controls (Supplemental Fig. 1). While the biological significance of this observation is unclear, these results do suggest a systemic or even coordinated role of C7 and RBP4 within and outside the central nervous system in ALS. Both complement^54^ and retinol signaling pathways^55–58^ have been proposed as therapeutic targets. These markers could act as a plasma proxy for such intervention.

### Multivariate Analysis

A major theme of this research was to identify a protein signature capable of separating individuals with ALS and controls. Diagnosis of ALS is performed by exclusion of other confounding diseases coupled with the eventual presentation of appropriate phenotypes. Consequently the need for sensitive and specific markers that can be measured objectively is sorely needed in the clinic. The first step in this process is the identification of markers and development of models that can differentiate individuals with ALS from control samples.

**Figure 4:**
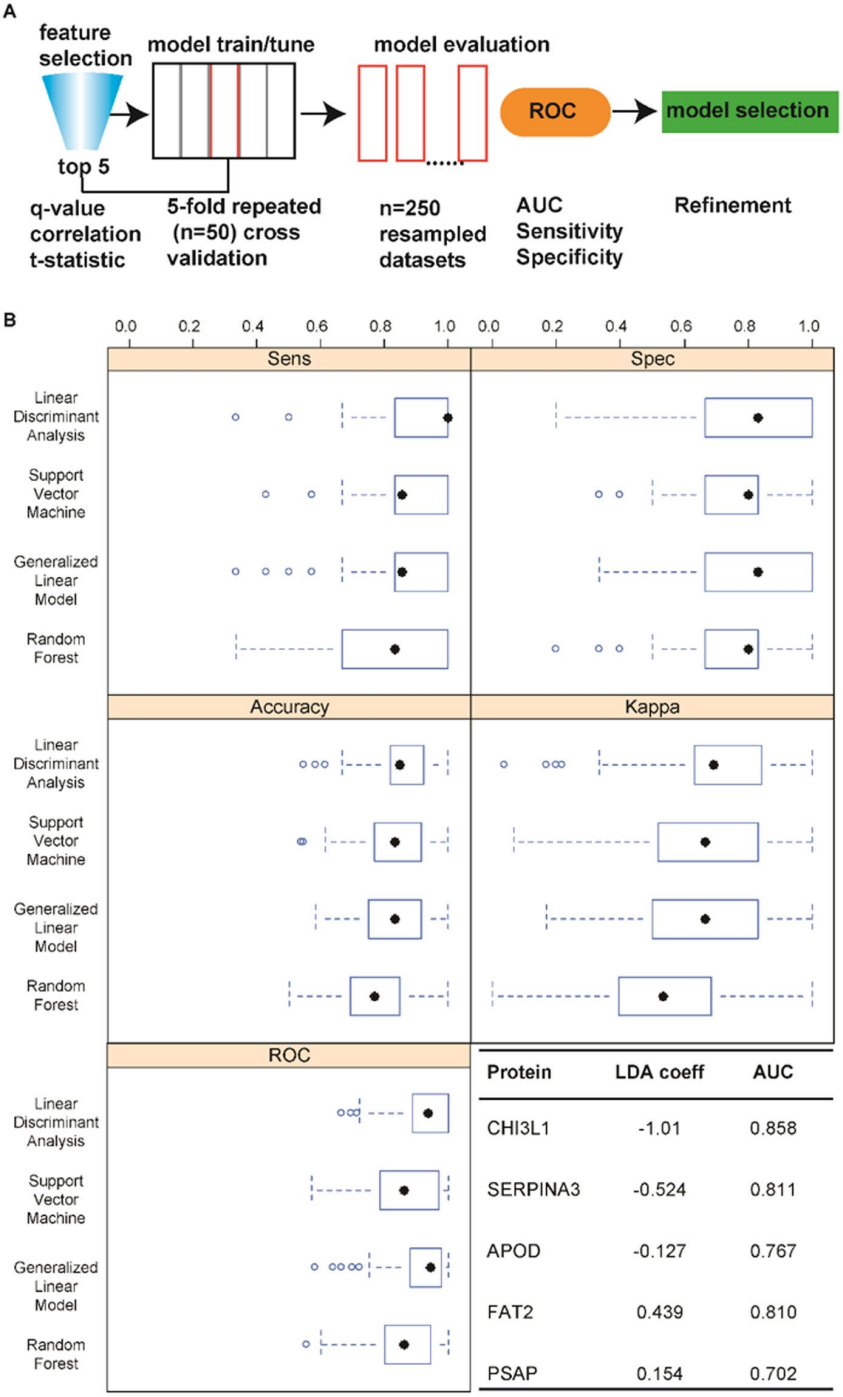
(**A**) An outline of the procedure used to develop and evaluate different algorithms for disease status prediction. (**B**) Comparison of the performance of 4 different machine learning algorithms on the resampled data using a repeated (n = 50) 5-fold cross validation approach. The coefficients of the LDA model and area under the curve rank the most important features for classification.

We investigated both the use of proteins in CSF and plasma for construction of a machine learning algorithm of diagnostic value. The procedure for the CSF data, outlined in Fig. 4A, consisted of feature selection, evaluation of the performance of different machine learning algorithms using repeated (n = 50) 5-fold cross validation, and statistical analysis of the results for each model on the resampled data sets. Five of the most significant uncorrelated proteins were chosen for inclusion in the model. The performance of four different algorithms was evaluated including linear discriminant analysis, support vector machine, random forest, and generalized linear model. Figure 4B shows box plots of the 5 standard metrics used for evaluation of classification algorithms on the resampled data sets (n = 250). Based on AUC, the linear discriminant analysis and GLM algorithms were identical and both significantly outperformed the other two models based on this metric (p < 0.05). Notably, the LDA model achieved higher sensitivity (p < 0.05) yet similar specificity (p > 0.05) compared to the GLM model. Finally the Youden Index (YI)^59^, which is a measure of the probability of making an informed decision (true positive or true negative), was compared using the resampled data. The Youden Index was highest (p < 0.01) for the LDA model (YI = 0.69; 63–0.83) followed by GLM (YI = 0.66; 0.5–0.83), SVM (YI = 0.66; 0.51–0.83), and RF models (YI = 0.55; 0.40–0.67). Due to its superior performance and interpretability the LDA model would be preferred. Based on the magnitude of the LDA coefficients and the area under the curve for individual proteins, the two most important proteins for classification were chitinase-3 like 1 and alpha-1-antichymotrypsin. Further data comparing the performance of the models can be found in Supplemental Fig. 2.

In addition to diagnostic markers, objective measures of prognostic value in ALS would be transformative. Currently disease progression/stage is assessed using a 12 question functional survey. Each question is scored (0–4) and the sum is referred to the patient’s ALS functional rating score^60^. ALS-FRS is the most widely used outcome measure of disease progression in ALS. The survey is often subjective and the scale is not linear in relation to the severity of functional impairment. Despite these limitations it is a measure of progression that has been used throughout clinical trials with success. Although it is well recognized that more quantitative metrics including markers related to specific biological pathways would greatly benefit the field.

**Figure 5:**
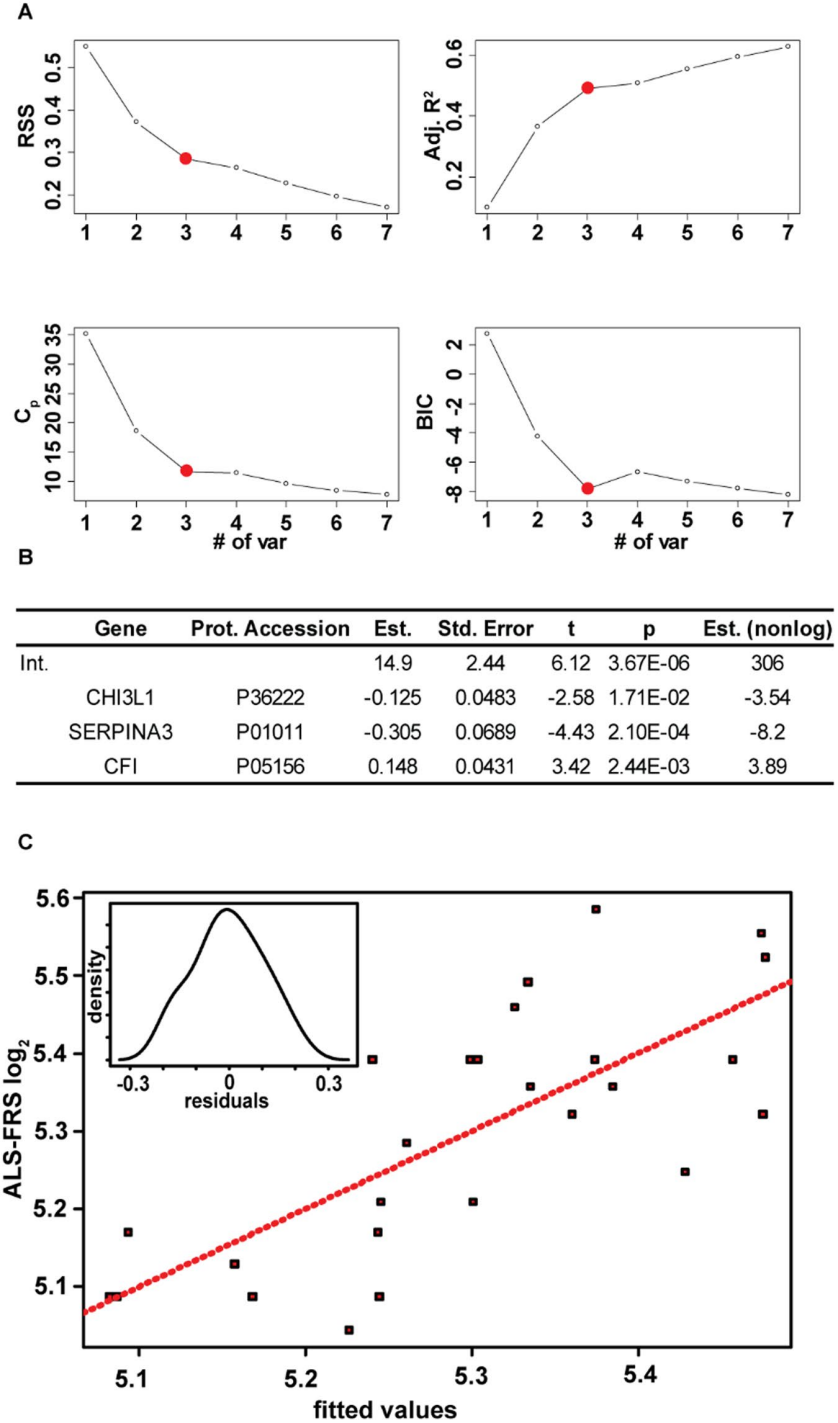
(**A**) Plots of the residual sum of squares, the adjusted r-squared value, Mallows Cp statistic, and the Bayesian Information Criterion (BIC) as a function of the number of proteins in the model. A three protein model was chosen. (**B**) Results from the regression analysis. (**C**) A plot of the ALS FRS scores as a function of the fitted values. Inset displays a density plot of the residuals.

The use of these markers for potential prognostic value was then investigated. A similar filtering method in which all proteins with a q < 0.05 were retained for evaluation was performed. After removing correlative features (Supplemental Fig. 3), we used multiple linear regression to develop a model of prognostic value. Figure 5A displays plots of 4 statistics that are commonly used to evaluate multiple linear regression models including the residual sum of squares, adjusted r-squared value, the Mallow’s C_P_ statistic, and the Bayesian Information Criterion, as a function of the number of variables included in the model. Based on previous recommendations involving the appropriate number of features to include in relation to sample size to minimize the risk of overspecification^61^, combined with the more marginal benefits observed with four or more variables, a 3 protein model was chosen. The model (F-stat = 9.03, p-value = 4.4e-4) explained 49% of the variation in the ALS-FRS scores. Figure 5B displays the results of the regression procedure and Fig. 5C shows a plot of actual vs fitted data. Density plot of residuals (Fig. 5C Inset) shows no deviation from normality (p = 0.8). Assessment of the residuals versus fitted and residual QQ plot reinforce the major assumptions for linear regressions were upheld (Supplemental Fig. 4). The three proteins in the model were chitinase-3 like 1, alpha-1-antichymotrypsin, and complement factor I. It is noteworthy that chitinase-3 like 1 and alpha-1-antichymotrypsin were identified as having both diagnostic and prognostic value. The exact role of chitinase-3 like 1 protein is unknown but it is believed to be heavily involved in immune response evidenced by its up-regulation in numerous inflammatory related diseases including obesity^62^, cancer^63,64^, and multiple sclerosis^65^. Rosa and co-workers^66^ showed upregulation of chitinase-3 like 1 transcripts in the spinal cord and motor cortex of sporadic ALS patients. Our results confirm a previous study that demonstrated that chitinase-3 like 1 protein levels were correlated with survival in ALS^67^. However, results described herein indicate that a multi protein model will improve upon the prognostic value of chitinase-3 like 1. Interestingly, all three proteins in the model are synthesized and secreted by the resident immune cells^68–71^ (microglia and astrocytes) in the brain which both further underscore the perturbation of the immune system in ALS^72^ and the non-cell autonomous view of motor neuron disease^73^.

While the majority of studies have focused on CSF as a fluid for biomarkers of diseases of the central nervous system, plasma remains an attractive biological fluid for detection of biomarkers due to its ease of sampling compared to a CSF draw (i.e., lumbar puncture). We chose to investigate the potential of a protein panel in plasma for disease classification. The procedure was similar to the one outlined in Fig. 4A except for utilizing a supervised feature selection method called recursive feature elimination (RFE)^38^. RFE avoids the repeated hypothesis testing associated with classical forward and backward selection techniques and is a preferred method when dealing with high dimensionality data. In addition, it aims to maximize accuracy based metrics of the model in comparison to the significance of individual features. Three models were evaluated including linear and nonlinear support vector machines and random forests. Models were chosen based on their innate ability to identify complex relationships in high dimensional data sets.

**Figure 6:**
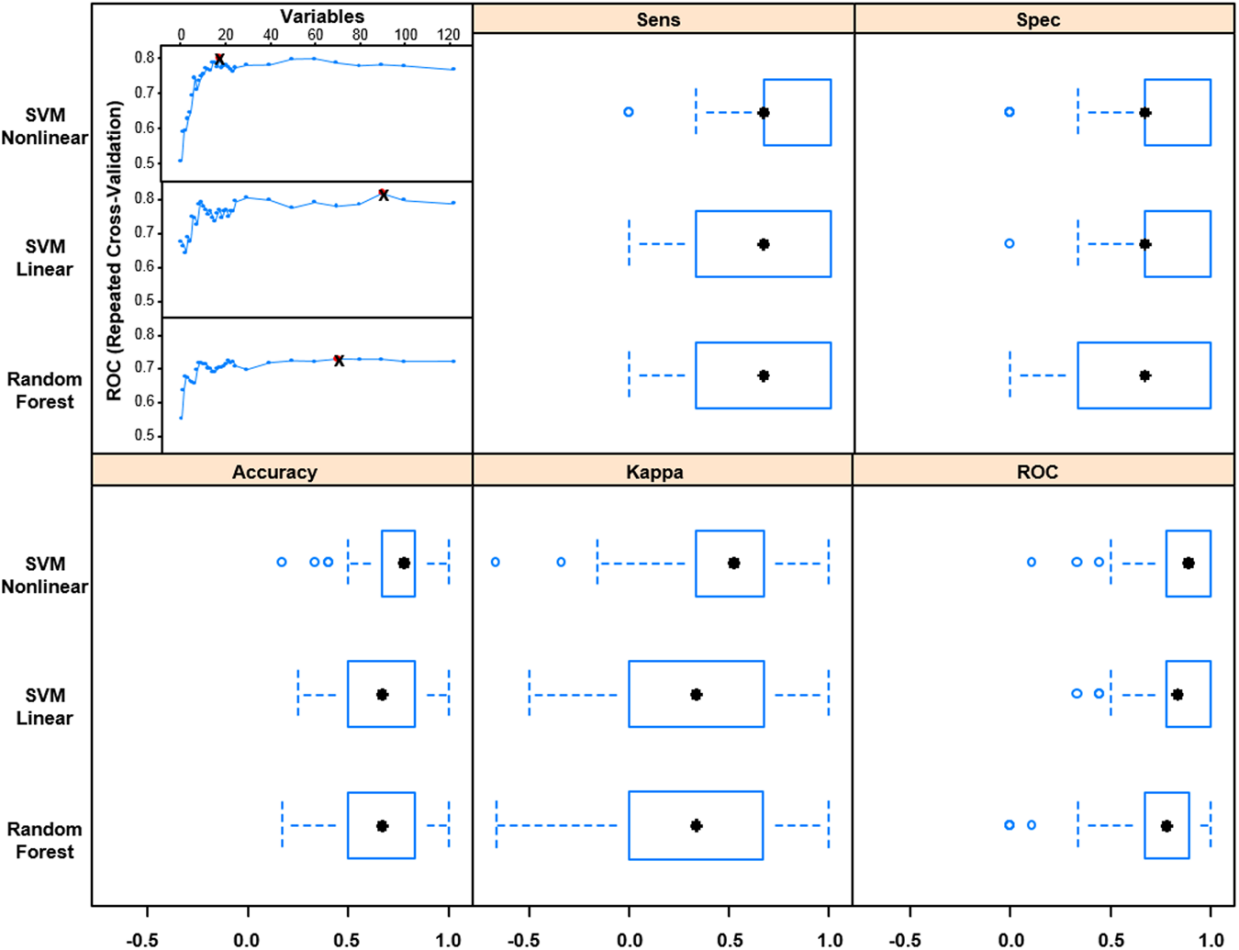
(**A**) The mean area under the curve of the resampled data sets is plotted as a function of the number of proteins used to create the model. (**B**) Comparison of the performance of the machine learning algorithms on the resampled data using the optimal number of proteins determined in (**A**).

Figure 6A displays plots of the mean area under the curve of the resampled data sets as a function of the number of proteins in each model. All three models showed a significant increase followed by a leveling of performance as a function of the number of proteins. This trend emphasizes the power of a multi protein panel for disease separation. The optimal number of features that maximized the classifier performance was 18, 90, and 70 for the nonlinear and linear support vector machine and random forest, respectively. Performance measures of the different models are compared in Fig. 6B. Using the area under the ROC curve as the gold standard the nonlinear and linear SVMs were equivalent in performance (p = 0.9) and both outperformed the random forest classifier (p < 0.05). Due to the identical performance, the nonlinear support vector machine would be favored due to its simplicity (18 vs. 90 proteins). The nonlinear svm achieved a median area under the curve of 0.89 and interquartile range from 0.78 to 1.0. The model could be further simplified by identifying the least number of features in the model within a certain threshold of the maximum AUC. By employing a 5% threshold, the model was reduced from 18 to 12 proteins with an insignificant effect on both the mean (p = 0.12) and median (p = 0.11) areas under the curve. A complete list of proteins used in the final model can be found in Supplemental Table 1. It is noteworthy that several of the proteins in the model participate in biological processes that are known to be altered in ALS including acute inflammatory response, proteolysis, complement activation, exocytosis, and blood coagulation. Several proteins were significantly correlated with the ALS FRS scores indicating the potential of plasma protein measurements for prognostic purposes. These proteins included ceruloplasmin, X-Pro dipeptidase, Antithrombin-III, and plasma kallikrein. Notably the latter two have opposing functions in the blood coagulation pathway.

### Validation of chitinase 3 like 1 protein and alpha-1 antichymotrypsin via targeted proteomics

Next we validated two of the more promising markers identified in a separate ALS CSF sample set using protein cleavage isotope dilution mass spectrometry coupled with targeted proteomics. The goal of this final experiment was two-fold: (1) to confirm the results from the discovery data in relation to differentiating a separate set of ALS from healthy samples; and (2) to evaluate the specificity of these markers to differentiate ALS from other neurological diseases. Surrogate peptides for proteins of interest were determined using an empirical refinement approach^31^. Peptides were evaluated based on uniqueness, abundance, and chromatographic performance. Candidate peptides were then screened for autosampler stability and digestion times were optimized (Supplemental Fig. 5A,B). Notably, protein depletion did not yield an appreciable increase in abundance of these targeted peptides yet was detrimental to quantitative precision (Supplemental Fig. 5C). Therefore, protein depletion was not performed in the preparation of this sample set. Two peptides per protein were chosen and respective SIL peptides analogues were synthesized and used to normalize response.

**Figure 7:**
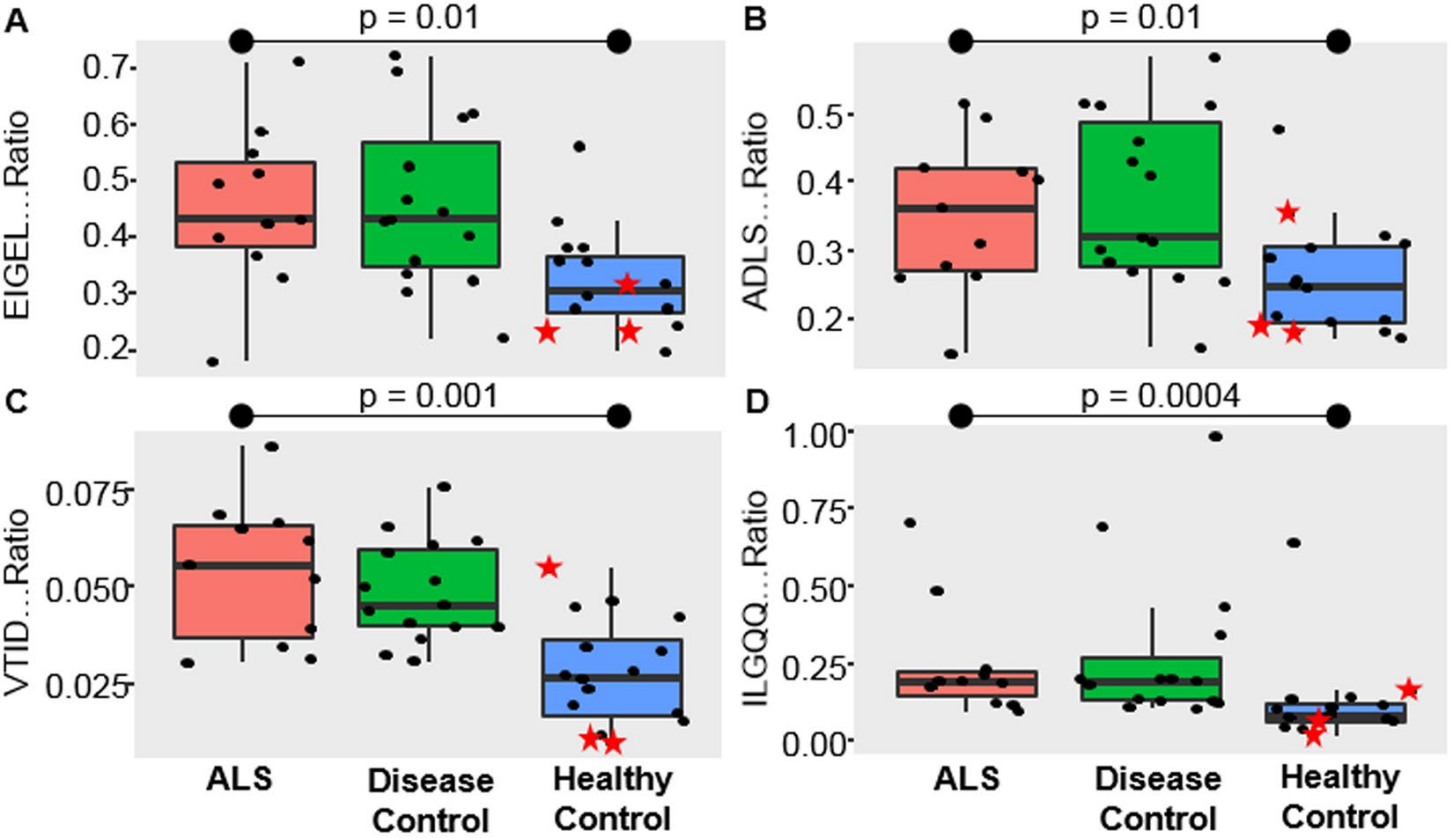
Boxplots of the abundance of the two peptide surrogates for alpha-1 antichymotrypsin (**A** and **B**) and chitinase-3 like 1 protein (**C** and **D**) across the groups. The 3 highlighted healthy samples in red (star) were new and previously not run in the discovery experiment.

The results confirm that alpha-1-antichymotrypsin (Fig. 7A,B) and chitinase-3 like 1 (Fig. 7C,D) are significantly elevated in the CSF from ALS patients compared to healthy. However, no significant differences in either protein were found when comparing ALS to other neurological diseases. These data support that the respective biological processes of these proteins (e.g., inflammation, microglia activation) are not unique to ALS but present in other neurological diseases. It could be argued that the clinical phenotype of other neurodegenerative diseases are often rather different from ALS and these markers could be used in conjunction with phenotype to aide diagnosis. However, significantly more research is needed to evaluate the capability of these markers to differentiate early on ALS and disease mimics^74^. In addition, these proteins could serve as an objective measure for future therapeutic interventions geared towards suppression of microglia activation in ALS and other neurodegenerative diseases.

## Conclusions

The ultimate goals of this study were to (1) identify biomarkers of diagnostic and or prognostic value and (2) further the understanding of the altered processes associated with ALS. To these ends, we performed shotgun proteomics on a set of matched CSF and plasma fluids from individuals diagnosed with ALS and healthy controls. The capability of a protein signature to separate ALS patients from controls was evaluated in both in CSF and plasma fluids using various machine learning algorithms coupled with Monte Carlo cross validation procedures. CSF proved most fruitful, with the development of a LDA model that achieved a medium area under the curve of 0.94 with an interquartile range of 0.88 to 1 on the resampled data sets. Two of the proteins used in the diagnostic classifier were also found to have prognostic potential and formed an MLR model that explained 49% of the variation in the ALS-FRS scores. While plasma is a distal fluid in ALS, the potential for disease separation based on a protein signature exists. We confirmed previous reports of significant enrichment of proteins involved in complement activation, acute phase response and retinoid signaling pathways. Furthermore targeted proteomics confirmed increased abundance of in a separate set of CSF samples compared to healthy yet no differences between ALS and disease controls. Future experiments will focus on targeted analysis of proteins identified in perturbed pathways using longitudinal sampling of biofluids.

## Electronic supplementary material

Dataset 1

Supplemental Information

## Data Availability

All raw data are freely available via public repositories as noted in Methods.
