# Supplementary material for: Machine Learning Reveals Protein Signatures in CSF and Plasma Fluids of Clinical Value for ALS
Source: Sci Rep. 2018 Nov 5;8:16334. doi: 10.1038/s41598-018-34642-x (PMC6218542; doi:10.1038/s41598-018-34642-x)
Supplement: Supplementary file 2 — Supplemental Information [file 41598_2018_34642_MOESM2_ESM.docx]

**Machine Learning Reveals Protein Signatures in CSF and Plasma Fluids of Clinical Value for ALS**

**Michael S. Bereman*,^1-3^ Joshua Beri^1^, Jeffrey R. Enders^3^, and Tara Nash^3^**

*^1^Department of Biological Sciences, North Carolina State University, Raleigh, NC 27695*

*^2^Department of Chemistry, North Carolina State University, Raleigh, NC 27695*

*^3^Center for Human Health and the Environment, North Carolina State University, Raleigh, NC 27695*

**Submitted to: Scientific Reports**

**Supplemental Information**

**Keywords:** Amyotrophic Lateral Sclerosis, Biomarkers, csf, proteomics

***Author for Correspondence**

Michael S. Bereman, Ph.D.

Department of Biological Sciences

Center for Human Health and the Environment

North Carolina State University

Raleigh, NC

Phone: 919.515.8520

Email: michaelbereman@ncsu.edu

**Supplemental Figure 1** Plots of the abundances of complement protein 7 and retinol binding protein 4 in plasma versus cerebral spinal fluid in ALS patients (first row) and controls (second row)

**Supplemental Figure 2** A dot plot comparing the performance of the different models. Below is a table with the mean differences  in area under the curve and significance of the models


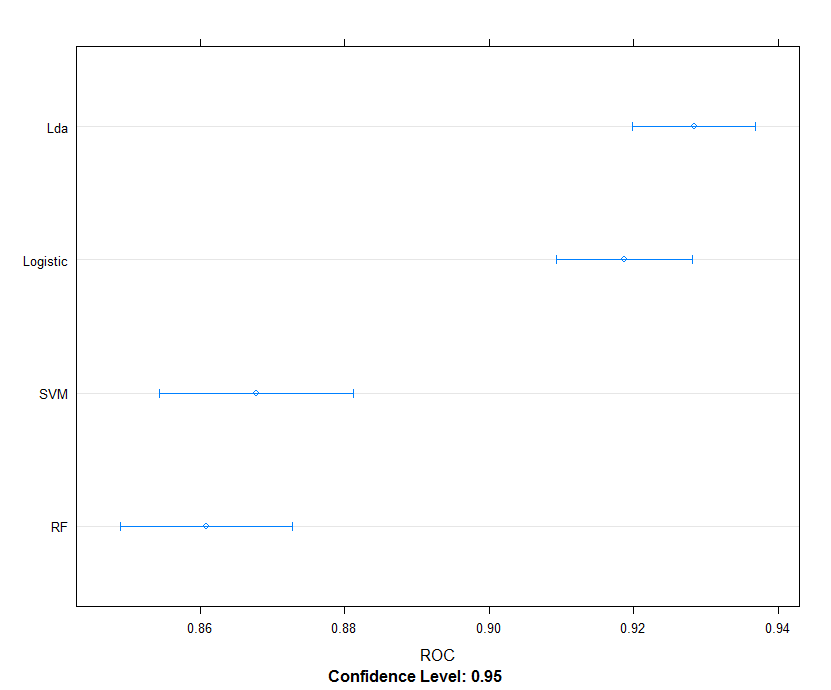


**Supplemental Table 1**: Pairwise comparison of the performance of each model using the CSF data


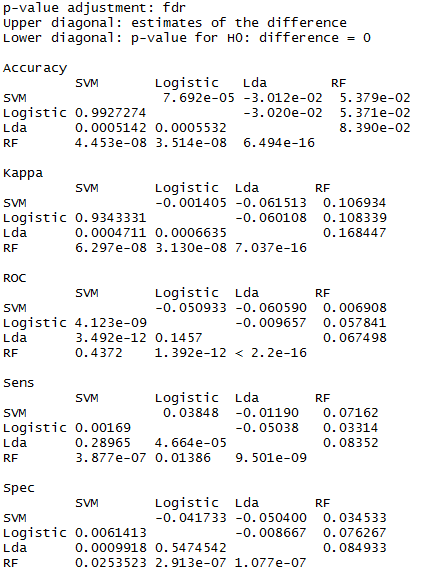


**Supplemental Figure 3** A plot of Pearson correlation coefficients amongst proteins found to be significant in CSF. Proteins labeled in red were removed from model building process


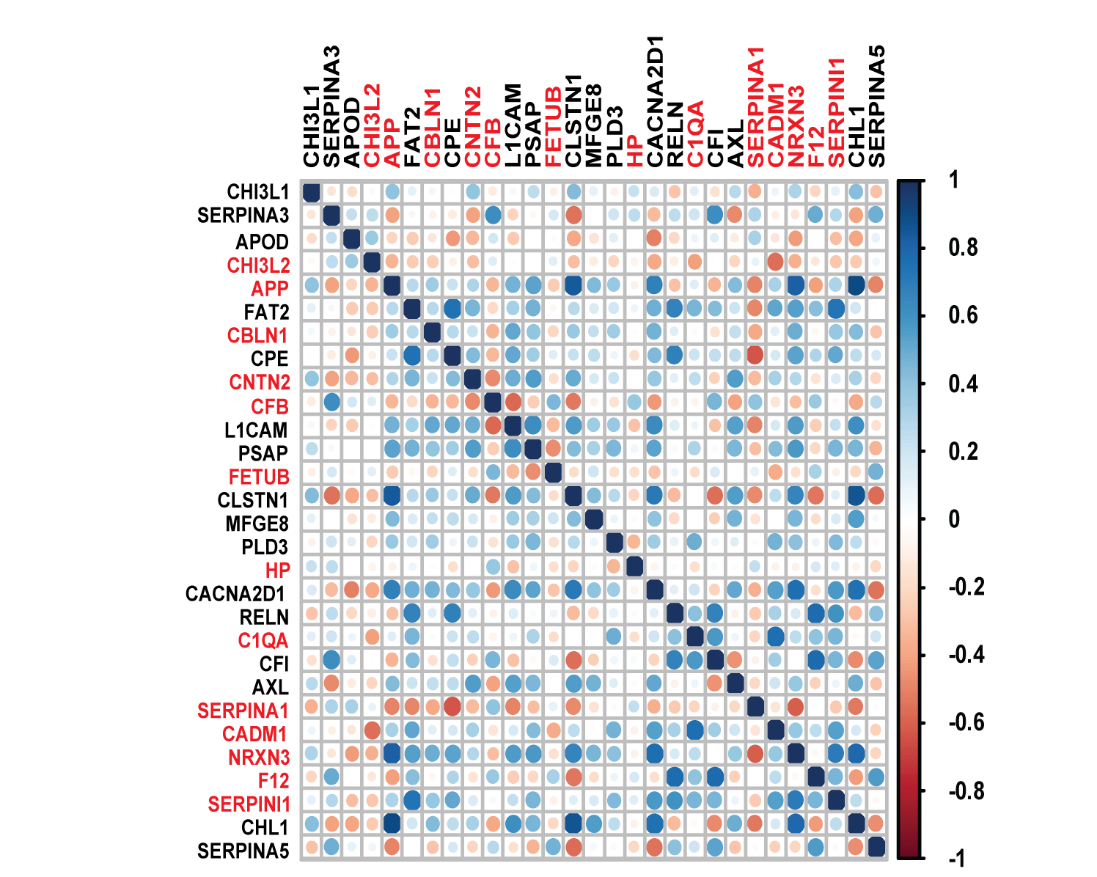


**Supplemental Figure 4** Residual versus fitted and QQ plot of residuals of the mlr model

**Supplemental Table** **2** list of the  plasma proteins used in the nonlinear support vector classifier

**Supplemental Figure 5** Evaluation of **A)** peptide stability **B)** digestion time and **C)** the need for protein depletion on abundance and precision.


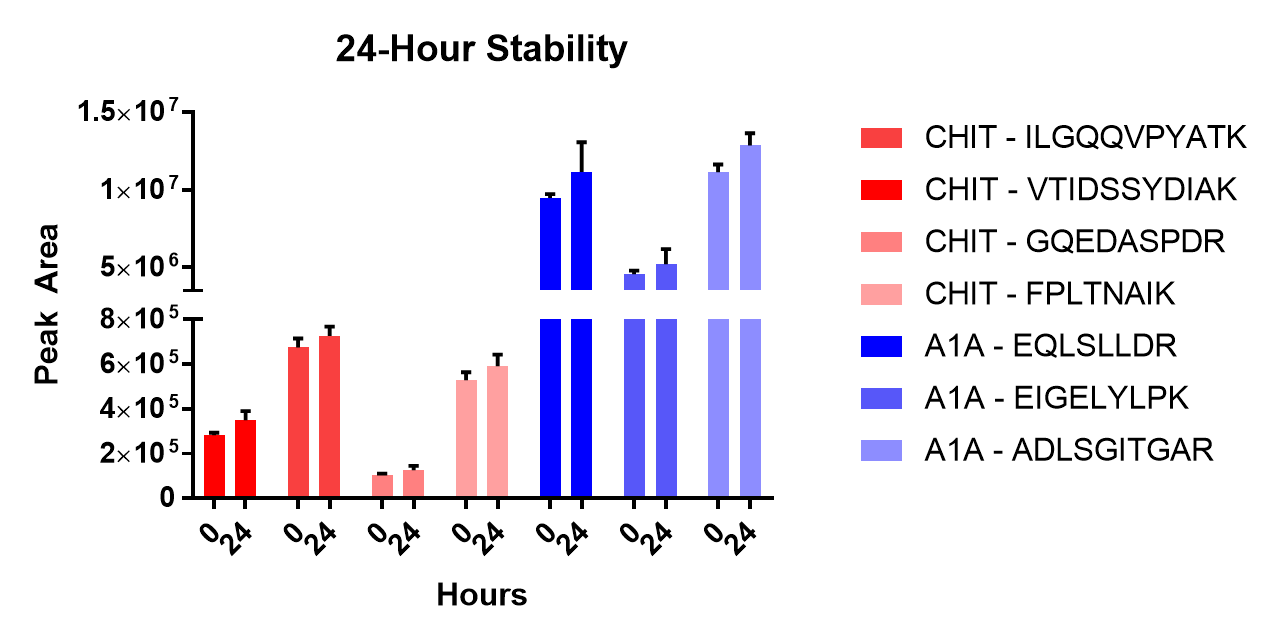

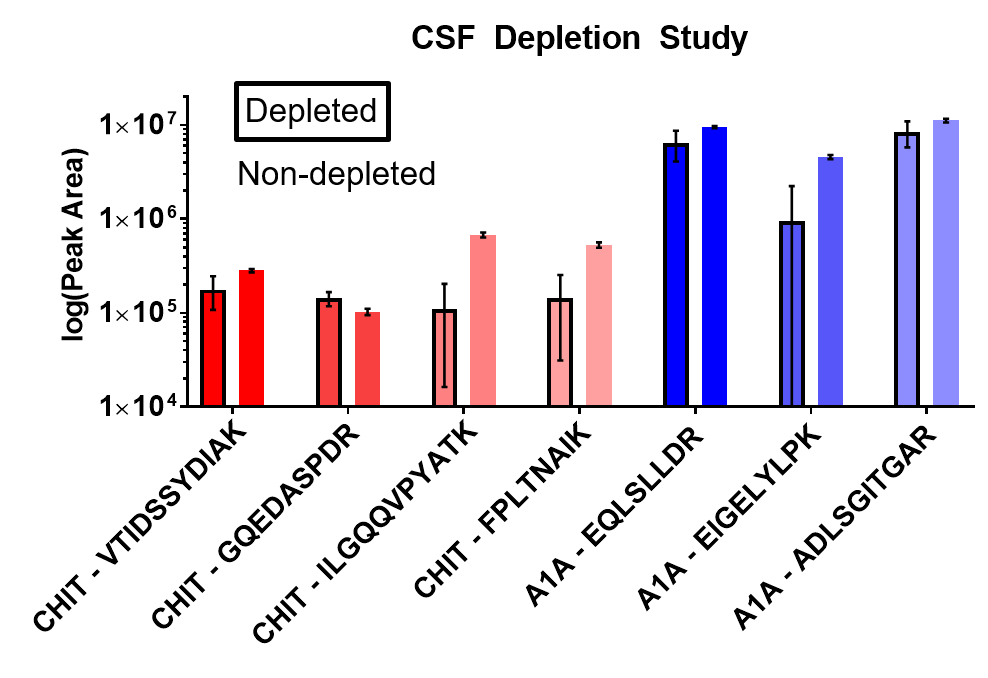

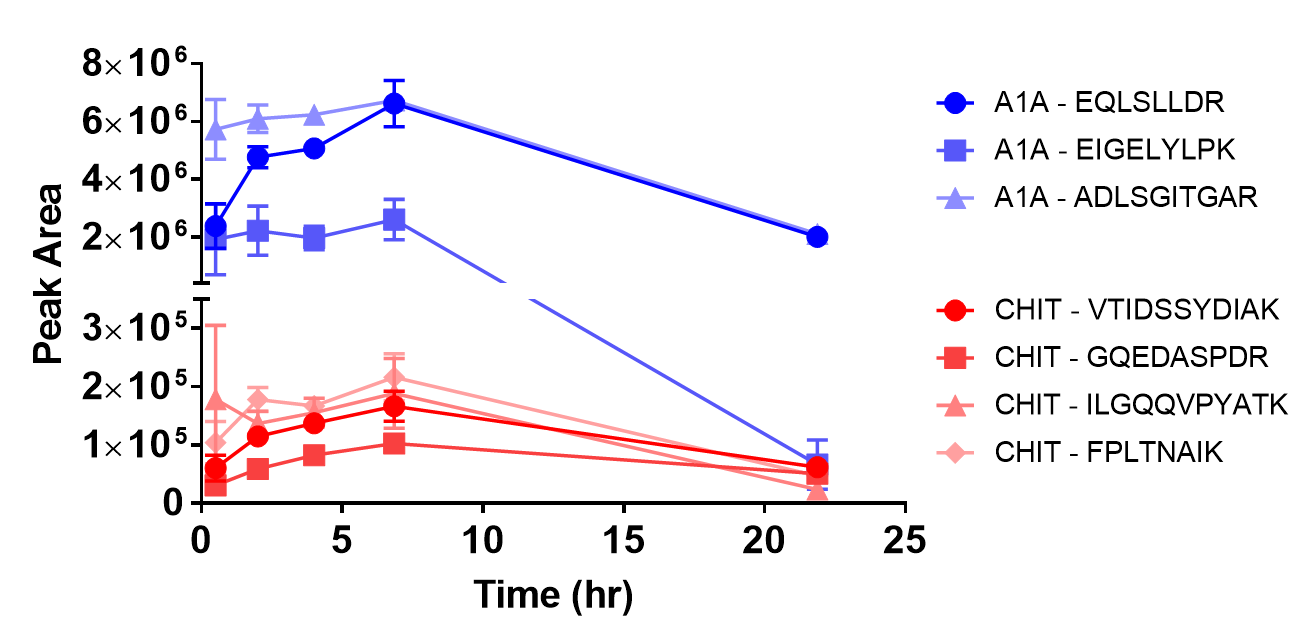


**A**

**C**

**B**
